# The metabolome as a biomarker of aging in *Drosophila melanogaster*

**DOI:** 10.1111/acel.13548

**Published:** 2022-01-12

**Authors:** Xiaqing Zhao, Forrest T. Golic, Benjamin R. Harrison, Meghna Manoj, Elise V. Hoffman, Neta Simon, Richard Johnson, Michael J. MacCoss, Lauren M. McIntyre, Daniel E. L. Promislow

**Affiliations:** ^1^ Department of Lab Medicine and Pathology University of Washington School of Medicine Seattle US; ^2^ Department of Genome Sciences University of Washington School of Medicine Seattle US; ^3^ Genetics Institute University of Florida Gainesville USA; ^4^ Department of Molecular Genetics and Microbiology University of Florida Gainesville USA; ^5^ Department of Biology University of Washington Seattle US

**Keywords:** aging, biomarker, drosophila, metabolomics, mortality

## Abstract

Many biomarkers have been shown to be associated not only with chronological age but also with functional measures of biological age. In human populations, it is difficult to show whether variation in biological age is truly predictive of life expectancy, as such research would require longitudinal studies over many years, or even decades. We followed adult cohorts of 20 *Drosophila* Genetic Reference Panel (DGRP) strains chosen to represent the breadth of lifespan variation, obtain estimates of lifespan, baseline mortality, and rate of aging, and associate these parameters with age‐specific functional traits including fecundity and climbing activity and with age‐specific targeted metabolomic profiles. We show that activity levels and metabolome‐wide profiles are strongly associated with age, that numerous individual metabolites show a strong association with lifespan, and that the metabolome provides a biological clock that predicts not only sample age but also future mortality rates and lifespan. This study with 20 genotypes and 87 metabolites, while relatively small in scope, establishes strong proof of principle for the fly as a powerful experimental model to test hypotheses about biomarkers and aging and provides further evidence for the potential value of metabolomic profiles as biomarkers of aging.

## Graphical abstract

Age‐specific metabolome‐wide profiles of *Drosophila melanogaster* are highly correlated with sample age. In this study, we build a metabolome clock to measure age acceleration, the difference between predicted sample age and chronological age, and show that this can predict mortality risk and lifespan outcome.

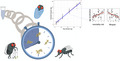

## 1 INTRODUCTION

Chronological age is the single greatest risk factor for functional deterioration, chronic diseases, and mortality (Harman, 1991). Several decades of research in laboratory organisms have led to the discovery of numerous hallmarks of aging and of evolutionarily conserved genetic pathways that can extend health span and lifespan (Kaeberlein et al., 2015). However, in real‐world settings, individuals vary in genetic and environmental background, and these factors mean that individuals of the same age can differ considerably in patterns of aging, even at quite young age (Belsky et al., 2015). To quantify this variation, to predict future trajectories of aging, and to identify the mechanisms that might underlie this variation, researchers have long sought predictive and prognostic biomarkers of aging (Baker & Sprott, 1988).

A diverse array of traits have been identified that associated with chronological age, including morphological and physiological features such as handgrip strength (Bohannon, 2015), vascular structure and function (Fedintsev et al., 2017), facial morphology (W. Chen et al., 2015), and molecular measures of variation in telomere length (Tsuji et al., 2002); transcriptome (Peters et al., 2015); proteome (Menni et al., 2015); metabolome (e.g., Yu et al., 2012); and epigenome, and this last typically measured through DNA‐methylation patterns (Hannum et al., 2013; Horvath, 2013).

Many of these features appear to be associated not just with chronological age but with functional measures of *biological* age. Markers of physiological deterioration across multiple organ systems are predictive of cognitive decline, self‐reported health, and facial aging (Belsky et al., 2015). Measures of immune function, oxidative stress, and inflammatory stress predict survival and recovery of centenarians after hospitalization (Martínez De Toda et al., 2019). Multiple biomarkers of blood cytometry and biochemistry are associated with physical and cognitive function, risk of age‐related diseases, and survival (Sebastiani et al., 2017). A focus of considerable recent research and genome‐wide measures of the methylome can not only predict chronological age but also the so‐called “age acceleration” (AA). AA refers to the difference between a subject's chronological age and their biological age. For example, an individual with a chronological age of 50 years, and a biological age of 55 years according to his/her methylome, is five years older than their chronological age that would have predicted and would have a concomitant increase in risk of morbidity and mortality.

Genome‐wide patterns of DNA methylation are associated with diverse aging phenotypes (Marioni et al., 2015), diseases of aging (Hannum et al., 2013; Horvath, 2013; Levine et al., 2015), and all‐cause mortality (e.g., Christiansen et al., 2016). While these so‐called epigenetic clocks have generated much enthusiasm in the aging community, they do have some limitations. In most cases, the studies are limited to cross‐sectional data. In many epigenetic clock studies, we do not know the underlying causes of these associations, though recent efforts are beginning to discern mechanisms (e.g., Horvath et al., 2015; Lu et al., 2020).

To address these challenges, here, we turn to the metabolome. The metabolome measures the complement of small molecules—metabolites—that are the outcome of metabolism and good representation of endophenotypes. Studies have shown that metabolite levels change with age (e.g., Yu et al., 2012) and have been used to detect physiological states such as frailty (Kameda et al., 2020), diseases such as cancer (Mayers et al., 2014), and age‐related degenerative processes in the nervous system (Wang et al., 2014) and the motor system (Swank et al., 2020). At least one recent study points to the ability of the metabolome to predict all‐cause mortality (Deelen et al., 2019). Like aging itself, metabolite levels are highly sensitive to genetic variation and to both intrinsic and extrinsic environmental variation (Hoffman et al., 2014; Jin et al., 2020; Kettunen et al., 2016).

In this study, we describe the development of a laboratory‐based model for a metabolome clock—one that allows us to measure not only survival but also functional aging, using a longitudinal design in a population with extensive genetic variation. To accomplish this, we take advantage of the *Drosophila* Genetic Reference Panel (DGRP), a set of inbred fruit fly strains derived from a wild population in Raleigh, NC (Mackay et al., 2012). These strains, which together offer a snapshot of current genetic variation within a single population, vary widely in diverse phenotypes (Mackay et al., 2012). Multiple studies have established extensive variation for metabolome profiles in the DGRP (Harrison et al., 2020; Hoffman et al., 2014), and recent work has mapped genetic variation for metabolite levels (Jin et al., 2020; Zhou et al., 2020). The fact that each strain within the DGRP is highly inbred allows us not only to obtain precise genotype‐specific estimates of lifespan and other demographic parameters but also to collect longitudinal measures of metabolome profiles. Although metabolome sampling requires sacrificing the fly, the inbred nature of each line is such that we are able to sample genetically identical individuals raised in a comparable environment at each age. Our previous studies with the DGRP have found that the metabolome is influenced by genotype, sex, age, and their interactions (Hoffman et al., 2014) but that when combined with the genetic structure of the DGRP, the metabolome can reveal otherwise hidden genetic variation for aging‐related traits, such as the response to dietary restriction (Jin et al., 2020) and oxidative stress (Harrison et al., 2020).

We follow adult cohorts of 20 DGRP strains chosen deliberately to represent the breadth of lifespan variation in the DGRP. We obtain estimates of lifespan and age‐specific mortality, fecundity, and climbing activity. We then ran our samples through a targeted metabolite profile that included 190 metabolites, representing a wide range of cellular pathways. We identified 87 metabolites that were consistently found in *Drosophila melanogaster* samples. We couple lifespan and related parameters with these functional data and profiles of 87 metabolites for each strain at multiple ages. We are able to show that activity levels and metabolome‐wide profiles are strongly associated with age, that numerous individual metabolites, such as kynurenine and putrescine, show a strong association with measures of aging, and that the metabolome provides a biological clock that not only predicts future mortality rates and lifespan but also has the potential to highlight specific mechanisms that underlie this clock.

## 2 RESULTS

### 2.1 Age at death

**FIGURE 1:**
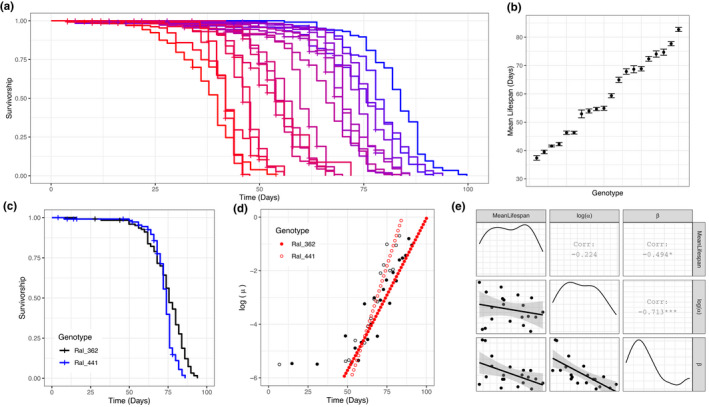
Variation in age‐at‐death among 20 DGRP strains. (a) Kaplan–Meier survival curves of 20 DGRP strains. Each line represents a different genotype. (b) Mean (± 1 SE) of lifespan for 20 DGRP strains. (c) Survival curves of Ral_362 and Ral_441. These two strains have similar mean lifespan (Ral_362 = 74.7 days; Ral_441 = 72.4 days), but they show significantly different survival curves (log‐rank test, *p* = 2 × 10^‒4^). (d) Instantaneous mortality risks of Ral_362 (filled dots) and Ral_441 (open dots). Black dots show measured mortality and blue dots show Gompertz–Makeham fitted mortality. Ral_362 has greater baseline mortality (log(*α*) = −11.5) compared to Ral_441 (log(*α*) = −15.9), but rate of aging is lower in Ral_362 (*β* = 0.115) compared to Ral_441 (*β* = 0.188). (e) Correlation among mean lifespan, log(α), and β. Shading around regression lines indicates 95% confidence intervals

We found substantial variation in age at death among the 20 DGRP strains that we examined (Figure 1a), ranging from a mean lifespan of 37 days in Ral_26 to 82 days in Ral_136 (standard error of mean lifespan <1.4 days, Figure 1b; log‐rank test, ($X_{19}^{2}$ = 3340, *p *< 2e‐16)). After fitting the Gompertz–Makeham (GM) model to each line, even genotypes with nearly identical mean lifespans had quite different baseline mortality rates *α* and rates of aging *β* (Figure 1c,d). While *β* appears to account for more variation in lifespan than does *α* (Figure 1e), we can explain most of the variation in lifespan by a linear model that includes both parameters (adjusted R^2^ = 0.91), and marginal impacts of both variables are both highly significant (*α*: *F*_1,17_ = 10.635, *p* = 0.0046; *β*: *F*_1,17_ = 184.9, *p* = 1.45 × 10^−10^). As pointed out by many prior studies, we also observe an inverse correlation between *α* and *β* (*ρ* = −0.794, *p* = 3.883 × 10^−5^), the so‐called Strehler–Mildvan relationship (Strehler & Mildvan, 1960).

We used GM parameters to estimate instantaneous mortality risk at specific ages for each line. While early‐life mortality estimates from *α* and *β* typically predict mortality rates well below the limits of detection (Promislow et al., 1999), in the DGRP lines we measured, mortality rates between the age of day 45 and day 60 were reliably estimated by the model and vary across genotypes. Day 45 and day 60 mortality were both highly correlated with mean lifespan (*ρ* = −0.968 and *ρ* = −0.955, respectively).

### 2.2 Functional traits as predictors of age at death

**FIGURE 2:**
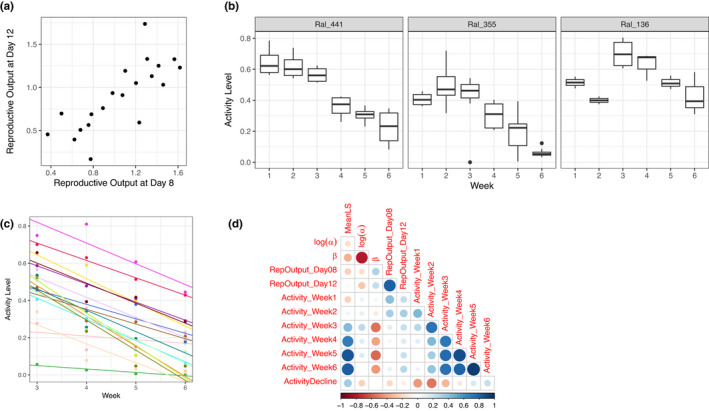
Variation in fitness‐related organismal phenotypes. (a) Variation in the square root of number of viable offspring produced by each female each day. Note that reproductive outputs at the age of day 8 and day 12 are highly correlated. (b) Examples of activity level change with age. Ral_441 represents a pattern of monotonic decline starting from the first week. Ral_355 and Ral_136 represent non‐monotonic change of activity level over age. Note that all three strains show monotonic decline in activity starting from week 3. (c) Linear regression of activity level over age between week 3 and week 6. Different colors denote different genotypes. (d) Correlation between age‐at‐death parameters and fitness‐related organismal phenotypes. Size of dots is proportional to absolute values of Spearman's *ρ*

We next asked whether measures of fecundity and activity levels at various ages were correlated with age at death parameters. We measured reproductive output and found substantial genetic variation among lines (Figure 2a) (*F*_18,19_ = 8.18, *p* = 1.55 × 10^−5^). Reproductive outputs at days 8 and 12 were strongly correlated across genotypes (*r* = 0.774, *p* = 6.127 × 10^−5^, Figure 2a). Neither day 8 nor day 12 fecundity was significantly correlated with lifespan, *α* or *β* (Figure 2d).

Activity levels measured by climbing ability varied significantly with genotype and age, the latter fitted as a factorial variable (genotype: *F*_18,89_ = 7.93, *p* = 6.07 × 10^−12^; age: *F*_5,89_ = 28.5, *p *< 2.2 × 10^−16^). We observed considerable variation among lines in age‐related change in activity during the first two weeks, with some increasing and others decreasing, after which most of the strains showed a strong monotonic decline in activity levels from week 3 to 6 (Figure 2b,c). Across genotypes, activity level was correlated with lifespan, and this relationship became stronger as flies aged: Week 3 activity level is marginally correlated with mean lifespan (*ρ* = 0.436, *p* = 0.056), whereas week 5 activity level is highly correlated with mean lifespan (*ρ* = 0.850, *p* < 2.2 × 10^−16^).

To capture the rate of decline in activity levels from weeks three to six, we constructed a linear model with activity predicted by age (fitted as a numeric variable) for each genotype and used the slope of the model as a measure of rate of activity level decline (Figure 2c). The rate of activity level decline was not correlated with mean lifespan (Figure 2d, *ρ* = 0.343, *p* = 0.178).

### 2.3 Age‐specific targeted metabolomics

**FIGURE 3:**
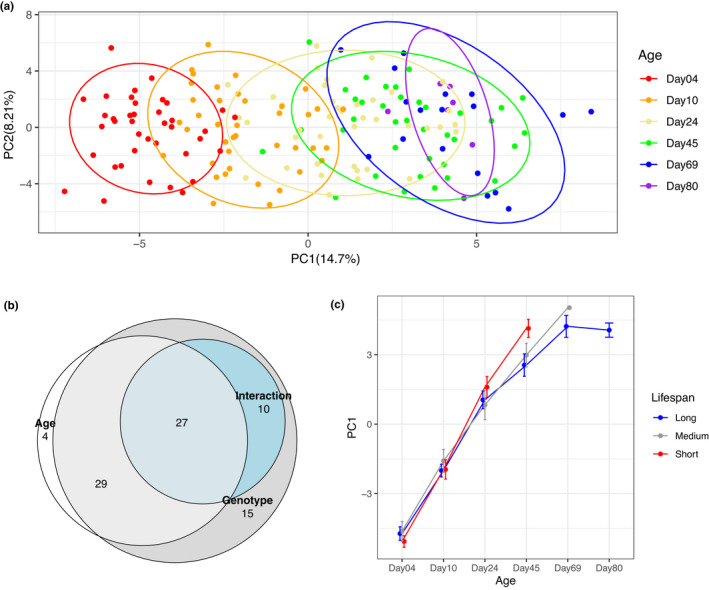
Overview of metabolomics data. (a) Principal components 1 and 2 of all samples, colored by sample age. Ellipses represent 90% confidence intervals. Note that PC1 separates samples of different ages. (b) Venn diagram illustrating overlap of metabolites with significant Age, Genotype, or Age × Genotype interaction terms. Areas shown in the figure are proportional to the numbers in each category. (c) Principal component 1 over sample age, stratified by genotype lifespan. Error bars show ±1 standard error

Across the 20 DGRP strains profiled here, we obtained measurements in all samples for 87 metabolites (Supplementary Table S7). Fitting a principal component model to the metabolome, we found that principal component 1 (PC1) provided a clear separation of samples by age (Figure 3a). The clearest separation among metabolome profiles was in animals 4 days to 45 days old (Figure 3a).

Using a linear model fitting age, genotype, and the interaction between the two as predictors of intensity for each metabolite, we found that a majority of measured metabolites were associated with age, genotype, and/or the interaction between the two (Table 1, Figure 3b). The large number of metabolites (more than 40%) associated with an age‐by‐genotype interaction underscores that the effect of age on the metabolome differs among genotypes and indicates that the metabolome might reflect biological age, rather than chronological age—a question we explore in detail below.

**TABLE 1:** Summary of result from the linear model: *Metabolite Level*=µ+*Age*+Genotype+Age×Genotype+ε

Fixed Effect		# of metabolites with FDR <0.01	% of total
Age	Increase	29	33.3
	Decrease	31	35.6
Genotype		81	93.1
Age × Genotype		37	42.5

### 2.4 Individual metabolites as predictors of age at death

**FIGURE 4:**
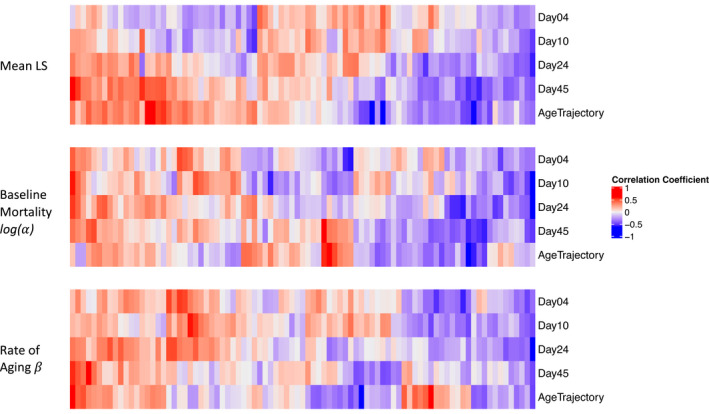
Individual metabolites as predictors of age at death. Correlation coefficients between age‐at‐death parameters and age‐specific metabolite levels or age trajectory of metabolite levels. Note that order of the columns is different for each of the three mortality parameters

We identified metabolites whose levels were associated with strain‐specific age‐at‐death parameters (Table 2, Supplementary Table S4). We also found that while some metabolites, such as kynurenine, were associated with mean lifespan, *α* and *β* throughout the life of the flies, most metabolites showed associations with lifespan and *α* and *β* at specific ages (Figure 4), with shifts in associations occurring between days 4 and 45. Additionally, for lifespan and *α*, the number of associated metabolites increased as age increased (Table 2). In contrast, the number of metabolites associated with *β* remained relatively constant from early to mid‐age (Table 2).

**TABLE 2:** List of metabolites significantly associated with age‐at‐death parameters (FDR<0.2)

Mean Lifespan	Day 4	Day 10	Day 24	Day 45	Age trajectory
	Kynurenate	Kynurenate	Kynurenate	4‐Guanidinobutanoate	Deoxycarnitine
			N1‐Acetylspermine	4‐imidazoleacetate	γ‐Aminobutyrate
				Agmatine sulfate	Phenyethanolamine
				Cytidine	Pterin
				Deoxycytidine	Putrescine
				Hippurate	
				N,N,N‐Trimethyllysine	
				Nicotinamide	
				Phenylalanine	
				Phenylethanolamine	
				Proline	
Log(α)	Day 4	Day 10	Day 24	Day 45	Age trajectory
		Cystathionine	2,4‐Dihydroxypteridine	4‐Guanidinobutanoate	
		N,N,N‐Trimethyllysine	Indole−3‐acetate	Agmatine sulfate	
		N‐α‐Acetyllysine	Kynurenine	Deoxycytidine	
			N,N,N‐Trimethyllysine	Guanine	
			N‐α‐Acetyllysine	Gaunosine	
				Histamine	
				Histidine	
				Kynurenine	
				N,N,N‐Trimethyllysine	
				N,N‐Dimethyl‐arginine	
				N‐α‐Acetyllysine	
				Nicotinate	
				Ornithine	
				Phenylethanolamine	
				Proline	
				Putrescine	
				Sebacate	
β	Day 4	Day 10	Day 24	Day 45	Age trajectory
	Glutamate	2‐Aminoisobutyrate	2,4‐Dihydroxypteridine	Kynurenine	γ‐aminobutyrate
	N,N,N‐Trimethyllysine	N,N,N‐Trimethyllysine	Acetylcholine	N,N‐Dimethyl‐arginine	N,N‐Dimethyl‐arginine
	Putrescine		Kynurenine	Ornithine	Pterin
	Tryptophan		N1‐Acetylspermine		
			N‐Acetylputrescine		
			Tyrosine		
					

This relatively small targeted profile of 87 metabolites has limited power to identify enriched pathways among the thousands of metabolic features that likely occur in *Drosophila* (Hoffman et al., 2014). Nonetheless, there are at least two pathways that stand out. In total, we queried 1,044 univariate associations predicting three mortality traits based on 87 metabolites measured at each of four ages (days 4, 10, 24, and 45). Among these associations, 46 relationships have a nominal *P*‐value less than 0.01, and of these, 11 are directly or indirectly associated with arginine‐ornithine metabolism (including dimethylarginine, ornithine, proline, glutamate, glutamine, putrescine, and N_1_‐acetylspermine), and 8 include association of metabolites linked with tryptophan metabolism (including tryptophan, kynurenine, and kynurenate).

Tracking metabolite levels over time allowed us to estimate the trajectory of individual metabolite levels across ages for each genotype. In particular, we constructed a linear model with metabolite levels predicted by age and used the slope of the model as a measure of the age trajectory of metabolite level. For a small number of metabolites, we found that their age trajectory was associated with mean lifespan and *β* (Table 2, Supplementary Table S5). Three of the five metabolites whose trajectories are associated with mean lifespan are not among the metabolites whose level at day 45 is associated with mean lifespan, suggesting that the trajectory provides additional information compared to age‐specific metabolite levels.

### 2.5 Metabolome as a predictor of age at death

We then asked whether the metabolome as a whole is predictive of lifespan. We categorized the twenty genotypes into long‐ (mean lifespan >65 d), medium‐ (50 d ≤ mean lifespan ≤65 d), and short‐lived (mean lifespan <50 d) groups and found that the first principal component of the metabolome varied significantly with age (*F*_1,175_ = 420.3, *p* < 2.2 × 10^−16^), lifespan group (*F*_2,175_ = 3.141, *p* = 0.0457), and their interaction (*F*_2,175_ = 12.948, *p* = 5.7 × 10^−6^) (Figure 3c). The metabolism profiles were similar across lifespan groups at days 4 and 10. However, at day 45, the metabolism of short‐lived genotypes resembles that of long‐lived genotypes at day 69 and 80 (Figure 3c). This underscores the possibility that the metabolome reflects the biological age and might be associated with lifespan and other age‐at‐death parameters.

**FIGURE 5:**
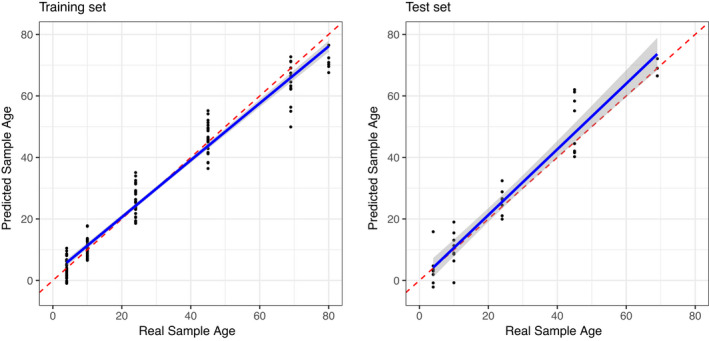
Metabolome is predictive of sample age. Both the training set (left) and test set (right) show elastic net regression predicted age vs. actual sample age. Red lines are the isometric line. Blue lines represent linear regression of the predicted sample age over real sample age. 80% of the samples were used to construct the model (left), and the model was used to predict age of the remaining 20% of samples (right). Predictions were repeated 100 times with different sets of training and test partitions. The figures shown here represent a case close to the mean R^2^ value

An elastic net regression supervised learning model demonstrated that the metabolome is highly predictive of chronological age (Figure 5, R^2^ ~ 0.9 in testing set). We compared several models that differ in the mixing percentage *λ*_1_ and regularization parameter *λ*_2_, and the best elastic net model turned out to be ridge regression (*λ*_1_ = 0), in which all of the metabolites contribute to the prediction. Given that the ridge regression model predicted chronological age with high accuracy across all genotypes where the majority had normal lifespans, we hypothesized that the predictions for chronological age for files of longer or shorter lifespan would reflect their biological rather than chronological age. Following existing literature (B. H. Chen et al., 2016), we calculated the difference between the predicted sample age and chronological age as a measure of age acceleration. If the predicted age is greater than the chronological age (i.e., age acceleration of samples is positive), then this strain is considered biologically older at that age than expected and would be expected to have a decreased lifespan and *vice versa*.

**FIGURE 6:**
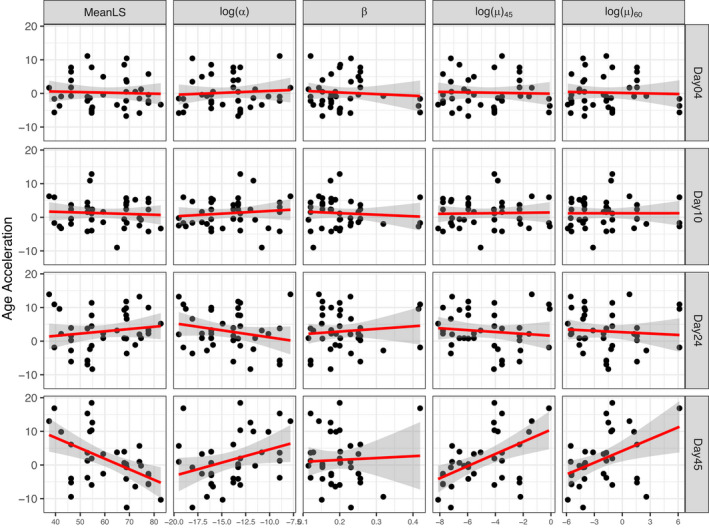
Age acceleration is correlated with demographic parameters. Red lines represent linear regression fit of data. Shading around red line shows 95% confidence interval of linear regression

Consistent with our previous observations, age acceleration predictions from days 4 and 10 did not show much variance across genotypes and did not predict mean lifespan or GM parameters. However, age acceleration predictions at day 45 were significantly associated with mean lifespan (*ρ* = −0.455, *p* = 0.0047, Figure 6) and baseline mortality (*ρ* = 0.333, *p* = 0.044). Additionally, age acceleration at day 45 was also predictive of the current mortality risk (*r* = 0.467, *p* = 0.0035) and mortality risk at day 60, (*ρ* = 0.402, *p* = 0.0136). In contrast, the rate of aging parameter *β* was not associated with chronological age (Figure 6).

## 3 DISCUSSION

Since the first epigenetic clock studies published in 2013 (Hannum et al., 2013; Horvath, 2013), enthusiasm has run high for clocks as biomarkers of aging. These papers, and the many other epigenetic clocks that have followed, underscore the tremendous potential for biomarkers of aging to predict aging and prognosis of disease. While the majority of studies on molecular aging clocks in human populations have focused on the epigenome, studies looking at metabolomic clocks in humans are becoming more common. Such studies have identified metabolites that are associated not only with chronological age but also with all‐cause mortality (Deelen et al., 2019) and age‐related clinical phenotypes (Jansen et al., 2021; Robinson et al., 2020). These results suggest that metabolome clocks can predict future health and suggest mechanisms for variation in age‐related decline. In a systems biological framework from genotype to phenotype, the epigenome is immediately downstream of the genome, while the metabolome is immediately upstream to the final phenotypes of interest, positioned to integrate upstream variation in genome, epigenome, transcriptome, proteome, microbiome, and intrinsic and extrinsic environmental factors. The work we present here not only supports the potential of the metabolome as a biological clock but also shows how the fruit fly can serve as a powerful model system to study the mechanisms underlying such biological clocks. These clocks can in turn be used to understand how genetic and environmental interventions work to increase health span and lifespan.

Taking advantage of the genetic variation in lifespan and age‐dependent stress resistance observed in the DGRP (Ivanov et al., 2015; Jin et al., 2020; Wilson et al., 2020), we demonstrate that the metabolome is highly predictive of age, and, moreover, of aging. Strains whose metabolome looks older than expected are shorter lived than those whose metabolome is younger than expected. We observed this pattern from middle‐aged measures of the metabolome but not from earlier samples. This is consistent with what we saw in age‐specific physiological phenotypes, where activity levels at mid‐ages (weeks 5 and 6) are more strongly associated with mortality metrics than are activity levels at earlier ages. This observation is also consistent with what we observe in studies of human biomarkers. For example, there is compelling evidence that gait speed is predictive of future morbidity and mortality, and like the metabolome in our study, the strength of this association increases with age (Montero‐Odasso et al., 2005).

Prior studies have shown that humans as young as in their 30s already exhibit extensive variation in rate of aging as measured by a suite of physiological and biochemical markers (Belsky et al., 2015). It is not clear, however, whether variation in rate of aging at such young ages is predictive of the eventual lifespan outcome. Our results suggest that even though age acceleration appears to vary at young ages, until mid‐life it may not be predictive of lifespan. This may be due to two concurrent mechanisms. First, evolutionary models suggest that genetic variation for aging should increase with age (Hughes & Charlesworth, 1994). Second, environmental variation in health status and endophenotypes accumulates with age (Bunning et al., 2020). The late‐age association between metabolome and lifespan that we observed could be due to a lack of underlying associations at early age. However, it might also reflect a lack of power to identify existing associations early in life due to low genetic variation, as predicted by theory.

The association between metabolome variation and aging is also notable given that previous studies had limited power to identify specific genes associated with variation in lifespan in the DGRP (Ivanov et al., 2015; Wilson et al., 2020). This study includes too few lines to carry out a genome‐wide association study. However, the results are consistent with recent studies showing that while few genes are associated with the lifespan response to diet restriction in the DGRP (Wilson et al., 2020), and numerous metabolites *are* predictive of that phenotype (Jin et al., 2020).

In carrying out this work, our hope was not simply to determine whether we could predict lifespan and mortality with metabolome profiles but also to use metabolome variation to identify potential mechanisms associated with variation in aging in the DGRP. The 87‐feature metabolite panel analyzed here is too small to provide sufficient statistical power to identify highly significant enrichment for specific pathways. Nonetheless, it is notable that two specific pathways are highly represented among those features associated with mortality measures at a nominal *p* < 0.01. First, we identified metabolites associated with tryptophan/kynurenine metabolism. Tryptophan is a precursor to serotonin and also has a degradation pathway leading to kynurenine and ultimately and to nicotinamide metabolism. There is considerable interest in the potential role both of kynurenine and nicotinamide metabolism as regulators of aging in multiple species including *Drosophila* (Oxenkrug et al., 2011). A second group of metabolites is also shown to be enriched in this analysis, including the amino acids arginine, ornithine and proline, and their related metabolites. Ornithine and arginine are precursors of polyamines, including putrescine and spermine, and molecules associated with mortality in our dataset. Previous work suggests that supplementation with polyamines can ameliorate the effects of brain aging in flies and mice (Gupta et al., 2013). Further studies with larger numbers of metabolites are needed, but taken together these results suggest that these two pathways may be causally associated with the considerable variation that we observe in rates of aging among the DGRP and that the brain may be an important regulator of this variation.

This study provides compelling evidence for the potential value of metabolomic profiles as biological clocks, but several caveats should be kept in mind. First, we chose our study population to maximize the available variation in lifespan. Doing this required us to perform prior lifespan analyses to determine which DGRP strains to include in the experiment, which may not be possible in most studies. In this design, we deliberately inflated the natural variance in demographical and physiological traits, and we expect this to have had a similar effect on the variation in the metabolome as compared to randomly selected strains. Future analyses based on random sampling of natural variation may require a larger number of genotypes to achieve the same statistical power. Second, we limit our analysis here to a targeted profile of 87 aqueous metabolites. The advantage of targeted metabolomic profiles is that they provide precise measures of features that are accurately defined. Global metabolomics on the other hand could provide a more comprehensive picture of metabolites associated with aging but incurs the trade‐off that the chemical structure of many of the features in global profiles is unknown. Moreover, we limit the analysis here to aqueous metabolites. Lipidomic variation has been associated with age in both humans and model systems (Wan et al., 2019; Wong et al., 2019), and thus, lipidomic analysis would be especially interesting with this population. Finally, at very late ages, as some genotypes die off, the experiment becomes unbalanced. We limit our analysis to ages when most of the genotypes are present, but future, larger‐scale studies might help to uncover metabolomic predictors of extreme lifespan.

## 4 EXPERIMENTAL PROCEDURES

### 4.1 Fly strains and fly husbandry

A set of 20 *Drosophila* Genetic Reference Panel (DGRP) (Mackay et al., 2012) strains were obtained from the Bloomington *Drosophila* Stock Center. All flies were maintained at low density on standard yeast–sucrose–glucose–cornmeal medium as described in (Harrison et al., 2020), in incubators at 24°C on a 12h: 12h light–dark cycle at ~50% humidity. All phenotypic assays and targeted metabolomic sampling were performed in two experimental blocks three months apart, with 11 DGRP strains in the first block and nine strains in the second.

### 4.2 Phenotypic assays

#### 4.2.1 Lifespan

Adults were collected during a 48‐h window after eclosion. Flies were then placed in bottles, in all cases here and elsewhere using SY10 medium as described in Linford et al. (Linford et al., 2013). Flies were allowed to mate for 24 h, and females were subsequently sorted under light CO_2_ anesthesia, with 25 females per vial, and five vials per strain. We chose to work with females, based on our earlier observation of greater variation in metabolomic profiles in DGRP females versus males (Hoffman et al., 2014). Vials were placed on trays in random order. Flies were transferred to fresh vials every other day without anesthesia, and the number of dead flies was recorded at the time of transfer, until all flies had died.

Mean lifespan of each strain was calculated as the restricted mean in the Kaplan–Meier model using the survival package in R (Therneau & Grambsch, 2000). We used maximum likelihood estimation implemented in the software package *WinModest* (Pletcher et al., 2000) to fit the Gompertz–Makeham parametric survival model to age‐at‐death data. The Gompertz–Makeham model, hereafter referred to as “GM,” is described by the equation

(1) $$\mu_{x}={\alpha e}^{\beta x}+M$$

Where µ*_x_* is the instantaneous mortality rate at age *x*, *α* is the “baseline” mortality (or the intercept in the standard Gompertz model where *M* = 0), *β* is taken as a measure of the “rate of aging,” and *M* is usually interpreted as a measure of extrinsic mortality (Missov & Lenart, 2013). Here, we fit *M* > 0 to correct for high early‐age mortality typical of fruit flies. The GM terms allow us to identify variation not only in how long each strain lives but also in the underlying mortality dynamics that determine lifespan.

#### 4.2.2 Age‐specific reproductive output

To measure reproduction, for a subset of flies at ages 8 and 12 days, vials were kept after transfer and left at room temperature for 16 days to allow eggs develop into adults, and the vials were then placed at −20°C. We then counted the number of adult offspring in each vial, and based on the number of females alive in the vial and the length of time interval between vial transfers, we were able to calculate the average number of viable offspring produced by each female in 24 h within each vial. Since females were separated from males by day 4, we did not examine fecundity beyond day 12, by which point sperm stores would be limited.

#### 4.2.3 Age‐specific activity level

A parallel set of females was obtained, following the same developmental protocols described above, to measure age‐specific activity and age‐specific metabolomic profiles. Climbing ability was assessed via a rapid iterative negative geotaxis (RING) assay (Gargano et al., 2005), details of which can be found in the Supplementary Information. For the first six weeks, adult fly climbing ability was assessed every week immediately following the transfer of the flies into vials of fresh food (removing dead flies). After the first six weeks, the majority of genotypes exhibited little climbing activity.

We used the non‐parametric Spearman’s *ρ* to evaluate correlations between age‐at‐death features (mean lifespan and GM parameters) and age‐specific fitness‐related phenotypes (reproductive output and climbing ability).

## 5 TARGETED METABOLOMICS

### 5.1 Sample collection

For metabolomic profiling, we collected cohorts of flies from each genotype at six time points (days 4, 10, 24, 45, 69, and 80). Samples of five females were collected into 1.5‐mL Eppendorf tubes and flash frozen in liquid nitrogen. About half of the strains did not survive to age 69 d, and only five strains survived to age 80 d. At each time point, when sample collection was complete, we selected five strains to have four biological replicates at all time points, five strains to have two biological replicates, and the remaining ten strains a single biological replicate, with a total of 182 samples for metabolomics. This design allowed us to assess the reproducibility of metabolomic data within an experiment that also included many ages and genotypes.

### 5.2 Sample preparation

To maximize our power to compare samples from different ages, we conditionally randomized metabolomics samples according to Ogut et al., 2019, where each batch contained samples from 4 to 6 strains, and each batch contained one replicate of all ages for each strain. Biological replicates were distributed across separate batches in the same manner.

Each frozen fly sample was homogenized in 200 µL H_2_O:PBS 9:1 in 2 mL Eppendorf tubes in a Next Advanced Bullet Blender for 5 min. We then added 800 µL methanol to homogenized tissues, vortexed for 10 s, incubated at −20°C for 30 min, and sonicated in an ice bath for 10 min. We then centrifuged the mixture at 14,000 rpm for 15 min at 4°C, transferred 600 µL of supernatant to a new Eppendorf tube, and completely dried under vacuum at 30°C for 2 h.

### 5.3 Liquid chromatography‐mass spectrometry (LC‐MS)

A ^13^C‐labeled internal standard was made by dissolving a metabolite yeast extract (Cambridge Isotopes Laboratory, ISO1) in 2.0 ml of water. About 150 µl of this stock was mixed with 850 µl of 0.1% heptafluorobutyric acid (HFBA) to make the solubilization buffer. The dried metabolite samples were solubilized in 50 µl 0.1% of this solubilization buffer, and 3 µl was injected onto the LCMS system. Samples were analyzed on a Vantage triple quadrupole mass spectrometer from Thermo Fisher using a Waters NanoACQUITY HPLC system, and a Waters ACQUITY UPLC M‐Class HSS T3 Column (100 Å, 1.8 µm, 300 µm × 100 mm) that was operated at a constant 25°C. Solvent A was 0.02% HFBA and 0.1% acetic acid in water. Solvent B was 0.02% HFBA and 0.1% acetic acid in acetonitrile. The gradient was 0%–25% B in 15 min, followed by 25%–100% B in 5 min. The flow rate was 5 ul/min. The precursor and product ion m/z values, their elemental compositions, collision energies, retention times, and ion adducts are listed in Supplemental Table S6. An amino acid standard mix was acquired between each batch as a system suitability test for the LCMS system. Three additional controls were acquired at the beginning and end of the entire acquisition—a blank containing 0.1% HFBA, a blank containing only a heavy labeled yeast metabolite internal standard, and a mixture of the targeted molecules (IROA standards from Sigma‐Aldrich). Chromatograms for each molecule were integrated in the program Skyline (Adams et al., 2020), which then created a CSV output file containing the chromatographic peak areas.

Targeted LC‐MS provided measures of 87 metabolites with known identity, with no missing data. One replicate sample of Ral‐440/Day 04 was removed because ~1/3 of metabolites was apparently abnormal. Data were log‐transformed to better achieve normality, and then each sample was centered and scaled to have a mean of 0 and standard deviation of 1.

## 6 STATISTICAL ANALYSIS

All statistical analyses were carried out using the open‐source statistics package R (R Core Team, 2020).

### 6.1 Linear Model

We used a mixed effect linear model implemented in the lme4 package to test for the effects of age, genotype, experiment block, and metabolomics batch on metabolite intensity:

(2) MetaboliteLevel=μ+Age+Genotype+Block+Batch+ε

where age is treated as a fixed effect, and genotype, experiment block, and metabolomics batch are treated as random effects.

Neither block nor batch explained a significant amount of variance for metabolite levels. Accordingly, the results shown here are from a simplified linear model,

(3) MetaboliteLevel=μ+Age+Genotype+Age×Genotype+ε

to identify metabolites whose intensity is significantly affected by age, genotype, or their interaction. Throughout, we used the Benjamini–Hochberg multiple testing procedure (Benjamini & Hochberg, 1995) to control false discovery rate (FDR).

To examine the association between individual metabolite levels at specific ages and demographic parameters, we used the linear model

(4) $${\mathrm{mx}}_{i,j,x}=\mu +\emptyset_{k}+\varepsilon$$

where *mx_i_*_,_ *_j_*_,_ *_x_* is the level of metabolite *i* in genotype *j* at age *x*, associated with Ø*_k_*, which is one of the three demographic parameters: mean lifespan (LS), intercept (*α*), and rate of aging (*β*).

We then examined the association between age‐trajectories of metabolite levels and demographic parameters. For each genotype, the age trajectories of metabolite levels were calculated as the coefficient of age in the linear model

(5) $${\mathrm{mx}}_{i,j,x}=\mu +Age+\varepsilon$$

where the coefficient indicates the direction and magnitude of metabolite level change over age. We used data only from the first four time points to calculate age trajectories, as some genotypes do not have samples at the later ages. Finally, to evaluate the correlation between age trajectory of metabolite levels and demographic parameters we used the linear model

(6) $$Metabolite\;Level\;Age\;Trajectory=\mu +\emptyset_{k}+\varepsilon$$

where Ø*_k_* refers to mean lifespan, *α*, or *β*.

### 6.2 Multivariate analysis

Principal component analysis (PCA) was performed on all samples using the prcomp function in R to observe the degree to which different ages and genotypes are distinguished by metabolome profiles.

To determine the degree to which the metabolome can be used to predict age, we used the elastic net regression model implemented in the glmnet package in R. Elastic net models create a penalized regression to avoid overfitting where the number of features is large relative to the number of samples, as in metabolomic data. Elastic net regression uses the loss function

(7) $$\min_{\left(\beta_{0},\beta \right)\in R^{p+1}}\frac{1}{2N}\sum_{i=1}^{N}{\left(y_{i}‐\beta_{0}‐x_{i}^{T}\beta \right)}^{2}+\lambda_{2}\left[\left(1‐\lambda_{1}\right){∥\beta ∥}_{2}^{2}/2+\lambda_{1}{∥\beta ∥}_{1}\right]$$

where *N* is sample number, *y_i_* is the age of a sample, and *x_i_* is the metabolome profile for that sample. The two tuning parameters include a mixing parameter (*λ_1_*) and a regularization parameter (*λ*_2_). If *λ*_1_ = 0, this is equivalent to Ridge regression (which includes all of the features in the model), and if *λ*_1_ = 1, it is equivalent to Lasso regression. The case of *λ*_1_ = *λ*_2_ = 0 is equivalent to ordinary least‐squares regression.

We randomly divided the 181 samples into training (80%) and testing (20%) sets, determined the appropriate parameters (*λ*_1_ was chosen among 0, 0.1…, 1.0; *λ*_2_ was chosen among 100 numbers spaced evenly between 0.000001 and 1, and 1000 numbers spaced evenly between 1 and 1000) within the training set using 5‐fold cross validation, and evaluated the R^2^ values in the test set. We repeated this process 100 times to make sure that the high level of predictability we observed was not due to chance.

To obtain a predicted age for each sample, we used leave‐one‐out cross validation (LOOCV), moving each sample one at a time from the dataset, and constructing a predictive model on the remaining data using the optimal mixing and regularization parameters determined by the training and testing procedure.

We calculated age acceleration for each sample as the difference between the predicted sample age and the real sample age. We then examined the correlation between metabolomic age acceleration values and each demographic parameter Ø*_k_* with the linear model

(8) $$\emptyset_{k}=\mu +\theta_{j,x}+\varepsilon$$

where *θ_jx_* is age acceleration of genotype *j* calculated based on metabolome at age *x*.

## CONFLICT OF INTEREST STATEMENT

The authors declare that they have no conflict of interest.

## AUTHORS CONTRIBUTIONS

D.E.L.P. conceived of the presented idea. X.Z., L.M.M., and D.E.L.P. designed and planned the experiment and all analyses. X.Z., F.T.G., B.H., M.M, E.V.H., N.S., R.J., and M.M. performed the experiment. X.Z. analyzed the data. X.Z., D.E.L.P., L.M.M., and B.H. wrote the paper. All authors discussed the results and contributed to the final manuscript.

## Supporting information

Supplementary Material

Table S1‐S7

## Data Availability

Raw mass spectrometry files and Skyline documents are available on Panorama Public (URL: https://panoramaweb.org/MetabolicClock.url). Data and code of statistical analyses are available on GitHub (URL: https://github.com/promislowlab/zhao2021).
