# Supplementary material for: The metabolome as a biomarker of aging in Drosophila melanogaster
Source: Aging Cell. 2022 Jan 12;21(2):e13548. doi: 10.1111/acel.13548 (PMC8844127; doi:10.1111/acel.13548)
Supplement: Supplementary file 1 — Supplementary Material [file ACEL-21-e13548-s002.docx]

**Supplementary Results**

*Age at Death*

Measures of age at death across 20 DGRP lines were carried out in two separate blocks. We found no effect of block on mean lifespan nor on Gompertz-Makeham (GM) parameters *α* and *β* (ANOVA, mean lifespan: *p* = 0.43; log(ɑ): *p* = 0.754; β: *p* = 0.905; M: *p* = 0.369). Accordingly, all subsequent analysis included combined data from both experimental blocks.

*Functional traits as predictors of age at death*

For fecundity, we first square-root transformed the average number of eggs produced by each female in 24 hours, which provided a good fit to a normal distribution (Shapiro-Wilks test *W* = 0.991, *p* = 0.222). Since there is no significant variance from replicate vials of each genotype (ANOVA *F_98,79_* = 1.071, *p* = 0.3778) we collapsed replicate vials of each genotype at each age. There was no significant difference in age-specific reproductive output between experiment blocks for either day 8 or day 12 measures (Day 8: *p* = 0.510; Day 12: *p* = 0.875), so data from both blocks were combined.

There was no significant difference in climbing ability between experiment blocks (*F*_1,89_ = 1.783, *p* = 0.185), therefore data from both blocks were combined.

The distributions of organismal phenotypic measurements are all normal across the 20 genotypes (reproductive output at day 8: *W* = 0.966, *p* = 0.667; reproductive output at day 12: *W* = 0.979, *p* = 0.927; activity level at week 3: *W* = 0.950, *p* = 0.374; activity level at week 4: *W* = 0.956, *p* = 0.460; activity level at week 5: *W* = 0.956, *p* = 0.564; activity level at week 6: *W* = 0.920, *p* = 0.146; activity level slope: *W* = 0.921, *p* = 0.154). Distributions of mean lifespan (*W* = 0.946, *p* = 0.314) and log *α* (*W* = 0.963, *p* = 0.606) are normal, and distribution of *β* is not normal (*W* = 0.848, *p* = 0.005). We used Spearman’s *ρ* to measure correlations between age-at-death parameters and organismal phenotypes.

*Targeted metabolomics*

Fitting each metabolite with a mixed-effects model of age, genotype, experimental block and metabolomics batch, we found that in most cases, age was the single greatest source of variation (Supplementary Figure 1), with genotype showing a more limited effect, and little to no effect of experimental block and metabolomics batch. Accordingly, we present a simpler univariate model here fitting only age, genotype, and the interaction between the two as predictors to intensity for each metabolite.

**Supplementary Material and Methods**

*Fly husbandry*

In preparation for the study, we expanded the population of each strain by setting up multiple bottles for three generations to remove maternal effects associated with stock maintenance. Prior to the study, we set up nine bottles for each strain, with approximately 100 females and 100 males in each bottle. Adult females and males were allowed to lay eggs in bottles and were removed after 48 hours. Excess eggs were removed from the surface of the media to ensure that each bottle contained no more than 300 eggs. Adults emerging from these bottles were used in the experiments described in this manuscript.

*Age-specific activity level*

To measure activity, we carried out a non-invasive climbing assay. Climbing ability was assessed via a rapid iterative negative geotaxis (RING) assay (Gargano *et al.*, 2005), which contained six vials, each of a different genotype, with 25 or fewer flies per vial. Videos were recorded as the RING apparatus was gently tapped down ten times. This minimizes the negative health effects potentially caused by a single large tap. This approach forced almost all the flies (99%, data not shown) to the bottom of the vial, followed by a negative geotaxis response. After the last tap down, the flies were given 4 seconds to climb, and the height above the level of the food of each fly in each vial was recorded in subsequent video analysis.

The distance climbed by each fly was normalized to control for variation in available vertical space in each vial due to variation in exact food height. Within each experiment block, the maximum calculated distance climbed is set as the available distance in the vial with the shortest empty space. We then take the ratio of adjusted distance climbed to adjusted maximal height as a measure of activity level. Vial was considered the unit of replication, with distance climbed as the mean value of all females in the vial.

*Targeted metabolomics sample collection*

For metabolomic profiling, we collected cohorts of flies over a 48-hour window and placed flies in bottles. Flies were allowed to mate for 24 hours and females were subsequently sorted using light CO2 anesthesia and placed into vials, with 25 females per vial, and six vials per strain. For five of the twenty strains, we collected another set of six vials of females and used them as biological replicates. We collected females for targeted metabolomics two days after we collected females for phenotypic assays, from the same set of rearing bottles for each strain.

We randomized vial order and transferred flies to fresh vials every other day without anesthesia, collecting samples from each genotype at six time points (days 4, 10, 24, 45, 69 and 80), using flies from one replicate vial per strain each time. After anaesthetizing these flies on ice, we transferred sets of five females into 1.5-mL Eppendorf tubes, and flash froze these samples in liquid nitrogen. We filled as many tubes of five flies as possible for each strain.

**Supplementary Figures**

Supplementary Figure 1. Variance partition of individual metabolites. Metabolites are ordered by total variance.

Supplementary Figure 2. Root mean square error (RMSE) over elastic net model parameters. Mixing percentage of 0 and regularization parameter around 5 yield the best-fitting model with the lowest RMSE in cross validation.
